# Revolutionizing Curcumin Extraction: New Insights From Non‐Conventional Methods—A Comparative Analysis of the Last Decade

**DOI:** 10.1002/jssc.70198

**Published:** 2025-07-08

**Authors:** Isabelle O. Torquato, Astrid Corrales, Cassamo U. Mussagy, Jorge F. B. Pereira, André M. Lopes

**Affiliations:** ^1^ Bioseparations and Nanoformulations Lab Department of Biotechnology Lorena School of Engineering University of São Paulo (EEL/USP) Lorena São Paulo Brazil; ^2^ Laboratorio de Desarrollo de Bioprocesos Sostenibles (Labisost) Escuela de Agronomía Facultad de Ciencias Agronómicas y de los Alimentos Pontificia Universidad Católica de Valparaíso Quillota Chile; ^3^ CERES Faculty of Sciences and Technology Department of Chemical Engineering Rua Sílvio Lima Pólo II ‐ Pinhal de Marrocos University of Coimbra Coimbra Portugal

**Keywords:** curcuminoids, eco‐friendly, environmental sustainability, purification, separation, solvents

## Abstract

Curcumin (CCM), derived from *Curcuma longa* L. rhizomes, holds significant pharmaceutical and biotechnological potential, driving extensive scientific research. Beyond its potent biological properties, the extraction of CCM has attracted considerable attention. Although numerous review articles have explored CCM extraction, a gap remains in the identification and comparison of methods aimed at improving these processes. This review seeks to address this gap by discussing efficient extraction methods that align with the principles of sustainability. It is essential to recognize that the effectiveness of both conventional and non‐conventional extraction methods depends not only on the techniques themselves but also on the optimal combination of these methods with suitable solvents. Conventional solid–liquid extraction methods often rely on volatile organic compounds which, despite their widespread industrial use, present several limitations, including low‐to‐moderate extraction yields, prolonged extraction times, the risk of thermal degradation or chemical alteration of the target molecule, and significant environmental impact. In contrast, non‐conventional, eco‐friendly methods, such as ultrasound‐, microwave‐, and enzyme‐assisted extraction, and supercritical fluid extraction, have garnered increasing attention. When combined with sustainable, neoteric solvents, these methods offer enhanced efficiency, selectivity, and environmental advantages. Future research should focus on optimizing and integrating these innovative extraction methods with green solvents to improve CCM yield, reduce environmental impact, and enhance scalability, ensuring alignment with sustainable practices in bioindustries.

**Abbreviations**
ABTS: 2,2'‐azinobis (3‐ethylbenzothiazoline‐6‐sulfonic acid)
BDMC: bisdesmethoxycurcumin
CCM: curcumin
CO_2_‐SFE: carbon dioxide supercritical fluid extraction
DES: deep eutectic solvents
DMC: desmethoxycurcumin
DMF: dimethylformamide
DMSO: dimethyl sulfoxide
DPCARB: *N,N*‐dipropylammonium *N’,N’*‐dipropylcarbamate
EAE: enzyme‐assisted extraction
ILs: ionic liquids
MAE: microwave‐assisted extraction
MNPs: magnetic nanoparticles
NADES: natural deep eutectic solvents
PLE: pressurized liquid extraction
SFE: supercritical fluid extraction
SLE: solid–liquid extraction
UAE: ultrasound‐assisted extraction
VOCs: volatile organic compounds

## 1 Introduction

The ongoing search for biologically active natural compounds, many of which are plant‐derived, represents a vast and dynamic field of research within pharmaceutical biotechnology. Among the phytochemicals of interest, curcumin (CCM) stands out as a prominent compound. This bioactive natural molecule, found in the rhizomes of the turmeric plant *Curcuma longa* (*C. longa*), exhibits a broad spectrum of biological properties, including notable therapeutic potential, thereby attracting growing interest n ot only from the scientific community but also from consumers at large [1, 2, 3].

**FIGURE 1:**
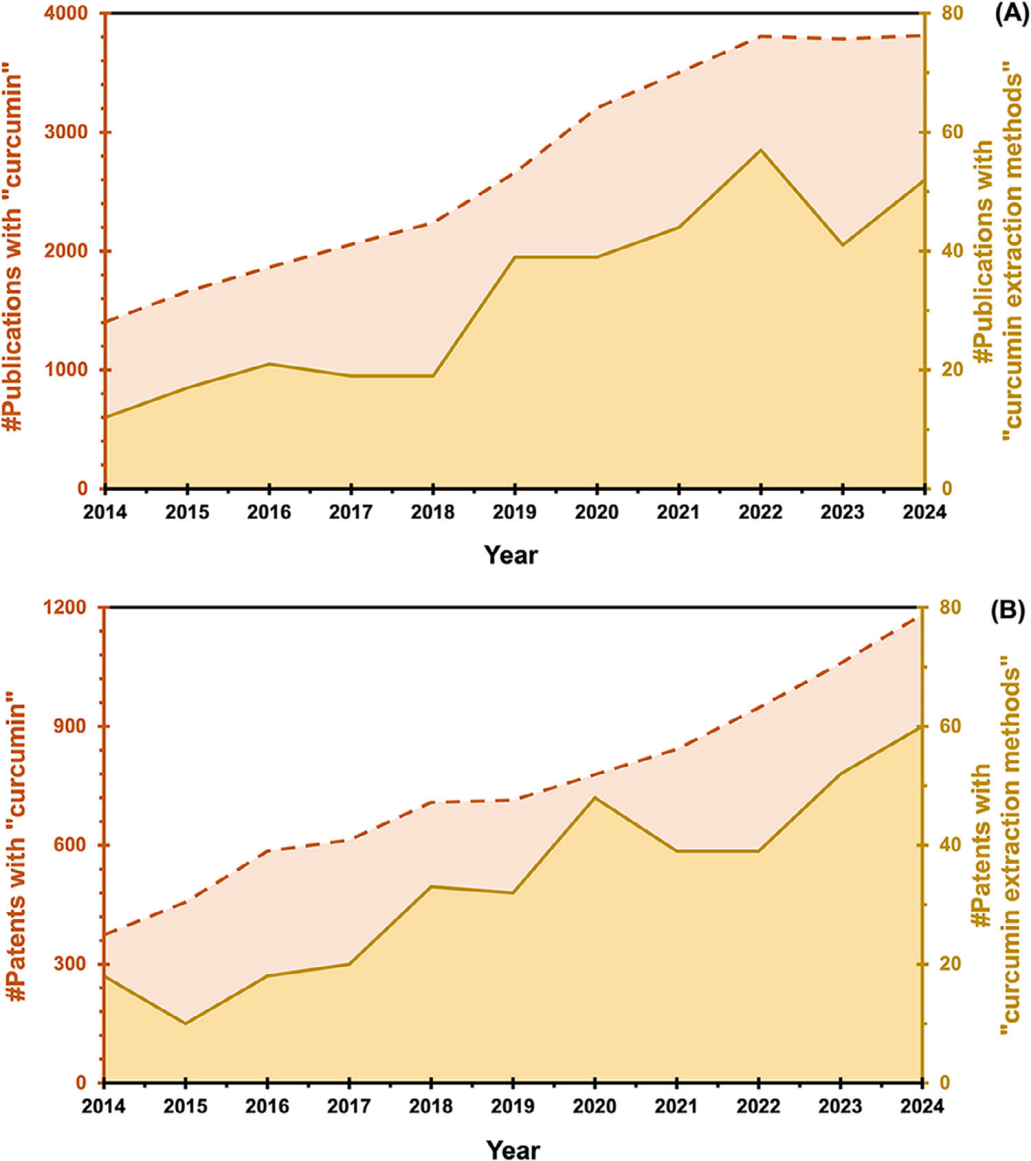
Number of scientific publications (**A**) and patents (**B**) retrieved using the keywords "curcumin" (dashed line) and "curcumin extraction methods" (continuous line) from 2014 to 2024. Data were collected from the Web of Science database on December 31^st^, 2024.

This growing interest is underscored by the significant increase in scientific publications related to “CCM”, totaling 29,992 publications. As shown in Figure 1A (dashed line), there were 1,404 publications in 2014, and 10 years later, this number rose sharply to 3,814 in 2024. However, despite the extensive research on this molecule, particularly regarding its medicinal, pharmacological, and biotechnological properties and applications, studies specifically focused on “CCM extraction methods” remain relatively scarce, with only 360 publications in total. Figure 1A (continuous line) indicates that the highest number of publications in this area occurred in 2022, with only 57 records. A total of 8,258 patents were identified between 2014 and 2024 using the keyword “curcumin”, with the highest number (1,182 patents) recorded in 2024 (Figure 1B). In contrast, the keyword “curcumin extraction methods” yielded significantly fewer results, totaling 369 patents over the same period. This category also peaked in 2024, with 60 patents recorded.

To fully understand the effective potential of CCM extraction, it is crucial to investigate the performance and efficacy of various extraction strategies, including both conventional and non‐conventional technologies. This research will not only advance knowledge in the field but also facilitate further R&D efforts. Conventional methods for extracting curcuminoids are typically conducted using solid–liquid extraction (SLE) strategies with pure or mixed solutions of volatile organic compounds (VOCs), mainly due to several key advantages. In general, the VOCs used for the solubilization of CCM are more lipophilic than water. Therefore, VOCs exhibit high solubility for curcuminoids, making them effective for extracting these compounds from *C. longa*. In addition, VOCs can easily permeabilize plant cell walls, enhancing the solubilization of curcuminoids in solvent and facilitates their recovery. The use of VOCs often leads to faster extraction processes and simpler purification strategies due to their high volatility and low boiling points, which allow for easy recycling and separation of the solvent from the curcuminoids. These processes are also easily scalable for industrial applications. Coupled with their competitive pricing, these advantages support the large‐scale production of curcuminoid extracts using such solvents. However, VOCs are associated with significant drawbacks. Certain classes of VOCs (e.g., hexane, methanol, and toluene) are toxic, flammable, and teratogenic, and their handling and disposal poses serious risks, particularly in terms of human and environmental safety. Given these concerns, the development and implementation of more sustainable and eco‐friendly extraction processes, using milder and less toxic solvents or employing process intensification strategies to eliminate the need for VOCs, offer considerable potential for the extraction and purification of curcuminoids [4, 5, 6, 7].

Non‐conventional extraction methods, such as ultrasound‐, microwave‐, and enzyme‐assisted techniques, as well as supercritical fluid extraction (SFE), have emerged as effective strategies when combined with the selection of optimal solvents. This synergy offers several advantages for curcuminoid extraction, including that of CCM. By integrating the most effective and appropriate solvents, these methods enhance extraction efficiency, reduce processing time, and preserve the integrity of bio‐compounds. Furthermore, they align with environmental sustainability and green chemistry principles by minimizing the use of harmful VOCs and reducing overall environmental impact. In addition, this approach enables precise control over extraction conditions, optimizing processes to achieve higher yields and superior curcuminoid quality. The aim of this review is to provide a comprehensive and critical analysis of the literature on CCM, with a particular focus on extraction processes used over the past decade (2014–2024). While the literature covers various aspects of CCM in depth, this review addresses general topics such as its natural source, chemical composition, bioactivity, and therapeutic properties. Specifically, our review is dedicated to evaluating the most commonly used methods for obtaining purified CCM, with special attention to new, environmentally sustainable approaches. Rather than focusing on the technical details of these methods, we emphasize their performance, as well as the advantages and limitations they present in extracting CCM. Through this discussion, we aim to offer a comprehensive and up‐to‐date understanding of CCM, from its origins to the latest extraction methods, highlighting environmentally conscious advancements in obtaining this valuable molecule.

## 2 General Aspects of Curcumin

### 2.1 Biological Origin and Plant Characteristics

The genus *Curcuma*, which comprises 133 species, is widely distributed across tropical and subtropical regions of Asia. In India, for example, Ayurvedic medicine, a holistic healing system, primarily relies on plant‐based remedies or extracts to treat various ailments. Several economically important species within the *Curcuma* genus are used, including *C. amada*, *C. aromatica*, *C. zedoaria*, and *C. caesia* [1].

*C. amada* is commonly used in culinary preparations such as pickles and salads and also serves as a stomach remedy and carminative. *C. aromatica* is used in medicine and the production of hygiene products, offering benefits such as relief from toothaches and swelling, deep cleaning, and the prevention of tartar buildup and bacteria that can cause gum problems like gingivitis. *C. caesia* rhizomes are applied externally to treat sprains and bruises, while *C. zedoaria* tubers are a source of starch that can be used in baby food [8, 9, 10].

**FIGURE 2:**
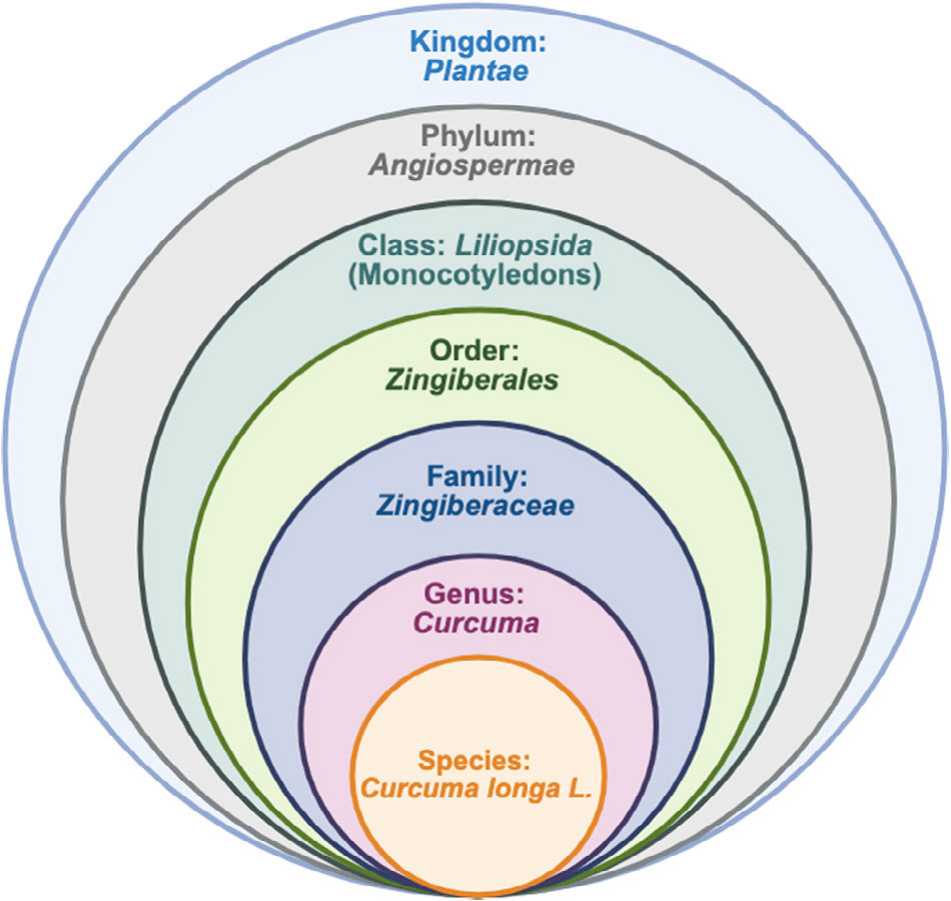
Scientific classification of *Curcuma longa* species.

Among these species, *C. longa* is widely recognized for its use as a spice (in the form of CCM powder) in food preparation [1, 9, 10, 11]. Scientifically named *Curcuma longa* Linnaeus (or simply *C. longa* L.), this species belongs to the order *Zingiberales*, family Zingiberaceae, and genus *Curcuma*, as depicted in Figure 2.

**FIGURE 3:**
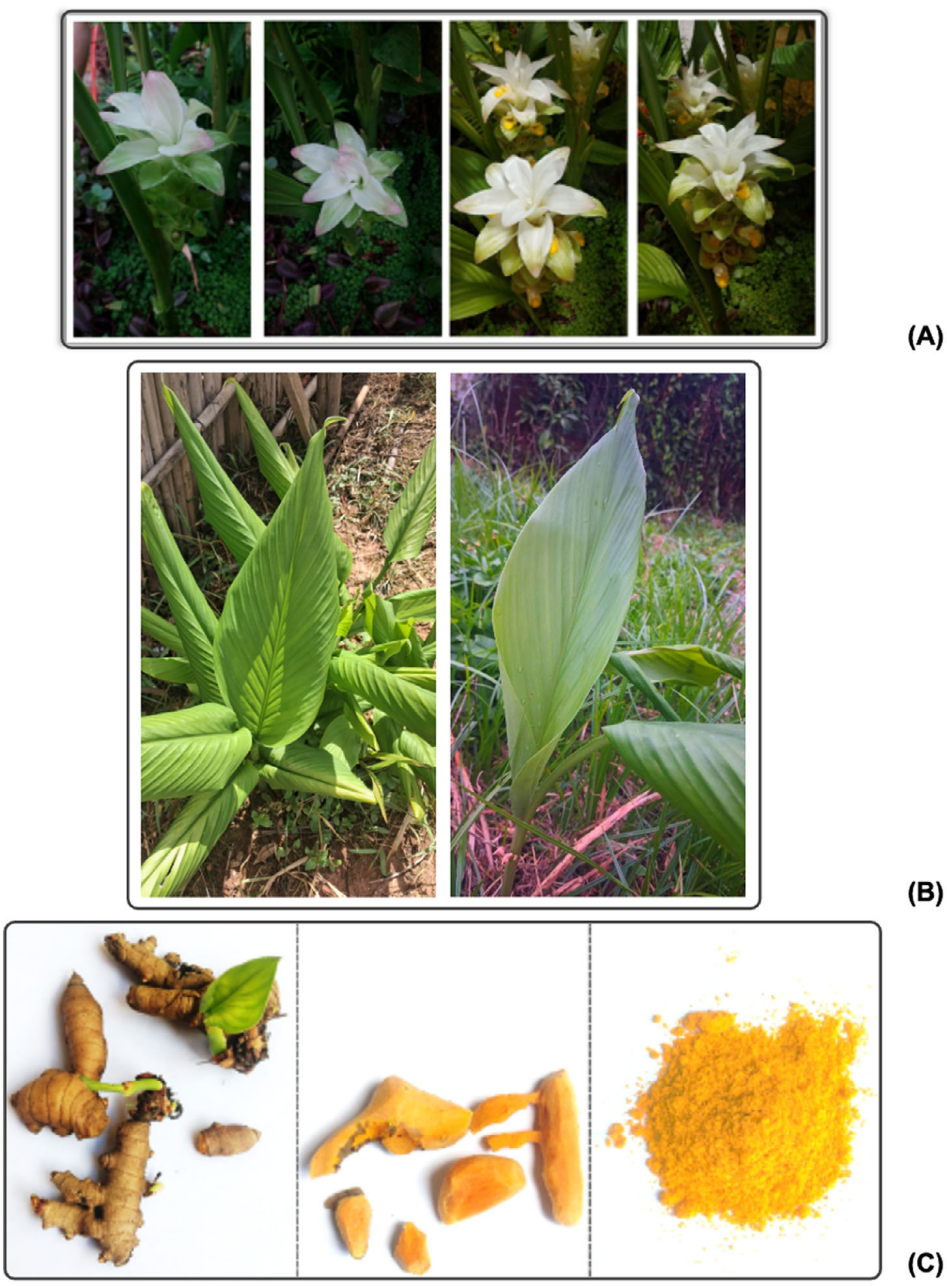
*Curcuma longa* plant showing prominent flowers (**A**) and large green leaves (**B**); rhizomes harvested, peeled, and processed into powder (**C**). Images were taken in the municipality of Itajubá, Minas Gerais State, Brazil (coordinates: 22°25′36.4116″ S, 45°27′10.728″ W) in February 2025.

The herbaceous plant *C. longa* typically grows to about 1 m in height, with long, broad green leaves, and is characterized by its bright orange tuberous rhizomes. Colorful bracts develop in terminal spikes, bearing lilac, white, or pink flowers that bloom during the summer months and encase small tubular flowers within the inflorescence structure (Figure 3A,B). While the flowers are visually appealing, the most valuable part of the plant is the rhizome, which is harvested and used in a variety of applications, ranging from culinary uses to the production of dietary supplements and medicines. The raw and dried rhizomes, along with the yellow–orange powder derived from *C. longa*, are shown in Figure 3C, illustrating the transformation from the natural form to the dried rhizome, and finally, to the powder. This yellow–orange powder is obtained by drying and grinding the plant's rhizomes [1, 12].

### 2.2 Chemical Composition

*C. longa* consists of approximately 60%–70% carbohydrates, 6%–13% water, 6%–8% protein, 5%–10% fat, 3%–7% dietary minerals, 3%–7% essential oils, 2%–7% dietary fiber, and 1%–6% curcuminoids. The golden‐yellow color of turmeric is primarily attributed to CCM, the main curcuminoid present in the plant [13].

The principal bioactive compounds in *C. longa* are diarylheptanoids, which include the curcuminoids. These compounds are characterized by two aromatic rings (aryl groups) connected by a seven‐carbon chain (heptane) with various substituents. The major curcuminoids are CCM, comprising 74.9%, desmethoxycurcumin (DMC) at 20.1%, and bisdesmethoxycurcumin (BDMC) at 4.9%. These compounds are structurally similar, differing only in the number of methoxy (─OCH_3_) groups present in their chemical structures [13, 14, 15, 16, 17]. In addition, *C. longa* contains 34 essential oils, with turmerone, germacrone, atlantone, and zingiberene being the primary constituents [18, 19].

Table 1 provides an overview of the curcuminoids, their molecular structures, and key physicochemical characteristics of CCM found in *C. longa*.

**TABLE 1:** Overview of curcuminoids: molecular structures, content, and physicochemical characteristics of curcumin (CCM), based on data from DrugBank [20] and Chemspider [21].

**Curcuminoids/Molecules**	Structures	**Content (%)**
**Curcumin (CCM)**	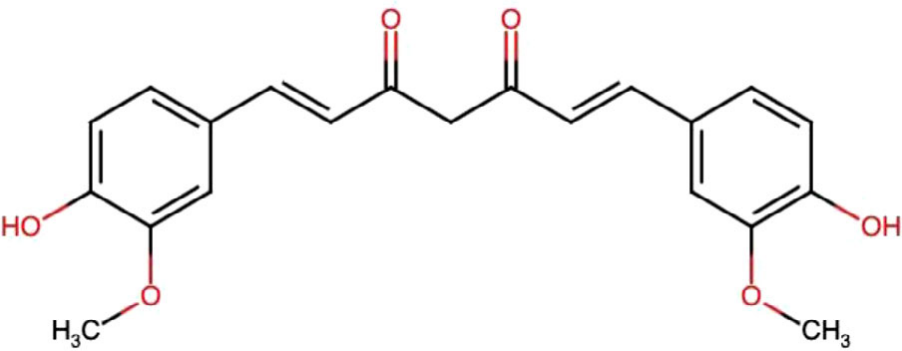	75
**Desmethoxycurcumin (DMC)**	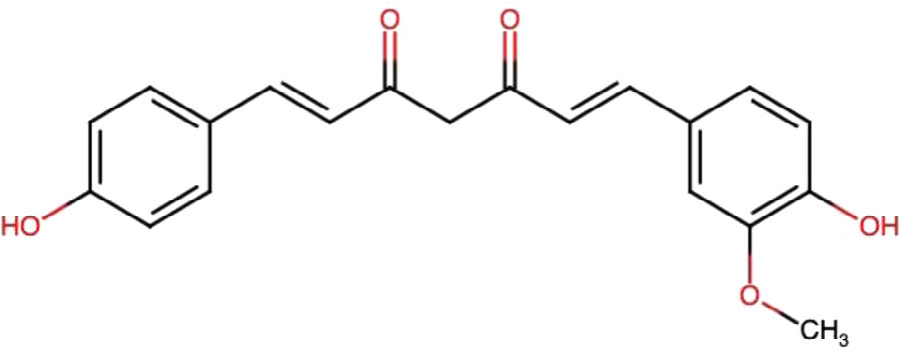	20
**Bisdesmethoxycurcumin (BDMC)**	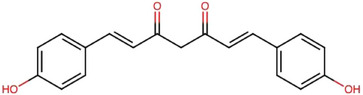	5

**Main physicochemical characteristics of CCM**	
**Systematic name**	(1E,6E)‐1,7‐Bis(4‐hydroxy‐3‐methoxyphenyl)‐1,6‐heptadiene‐3,5‐dione
**Other names**	Turmeric (referring to the aromatic rhizome of the Asian plant *C. longa*, a member of the ginger family, the powdered form is widely used as a condiment, particularly in curry powder)
**Molecular formula**	C_21_H_20_O_6_
**Molar mass**	368 g/mol
**Melting point**	183°C
**Color and aspect**	Yellow‐colored crystalline polyphenol
**Log** ***P***	3.2–4.1
**Solubility**	5.75 µg/mL in water; and 25 mg/mL in dimethyl sulfoxide
**Density**	1.3 ± 0.1 g/cm^3^
**Stability**	Stable under normal conditions but light‐sensitive and incompatible with strong oxidizing agents
**Bioactivity**	Exhibits antitumor, anti‐inflammatory, and antioxidant
**Market applications**	Used in herbal supplements, cosmetics, food flavorings, and food colorings

### 2.3 Bioactivity and Therapeutic Properties

CCM has been used by healthcare professionals to address a wide range of conditions, including diabetes, high cholesterol, inflammation, diarrhea, liver disorders, and asthma. Evidence also suggests that CCM is beneficial in the treatment of acne, joint pain, eczema, tonic and acute allergies, wound healing, and in supporting mood regulation, blood sugar levels, and immunomodulation. Human clinical trials have demonstrated that CCM is both effective and safe, and it has been classified by the U.S. Food and Drug Administration (FDA) as “Generally Recognized as Safe” (GRAS) [1, 22, 23, 24].

**FIGURE 4:**
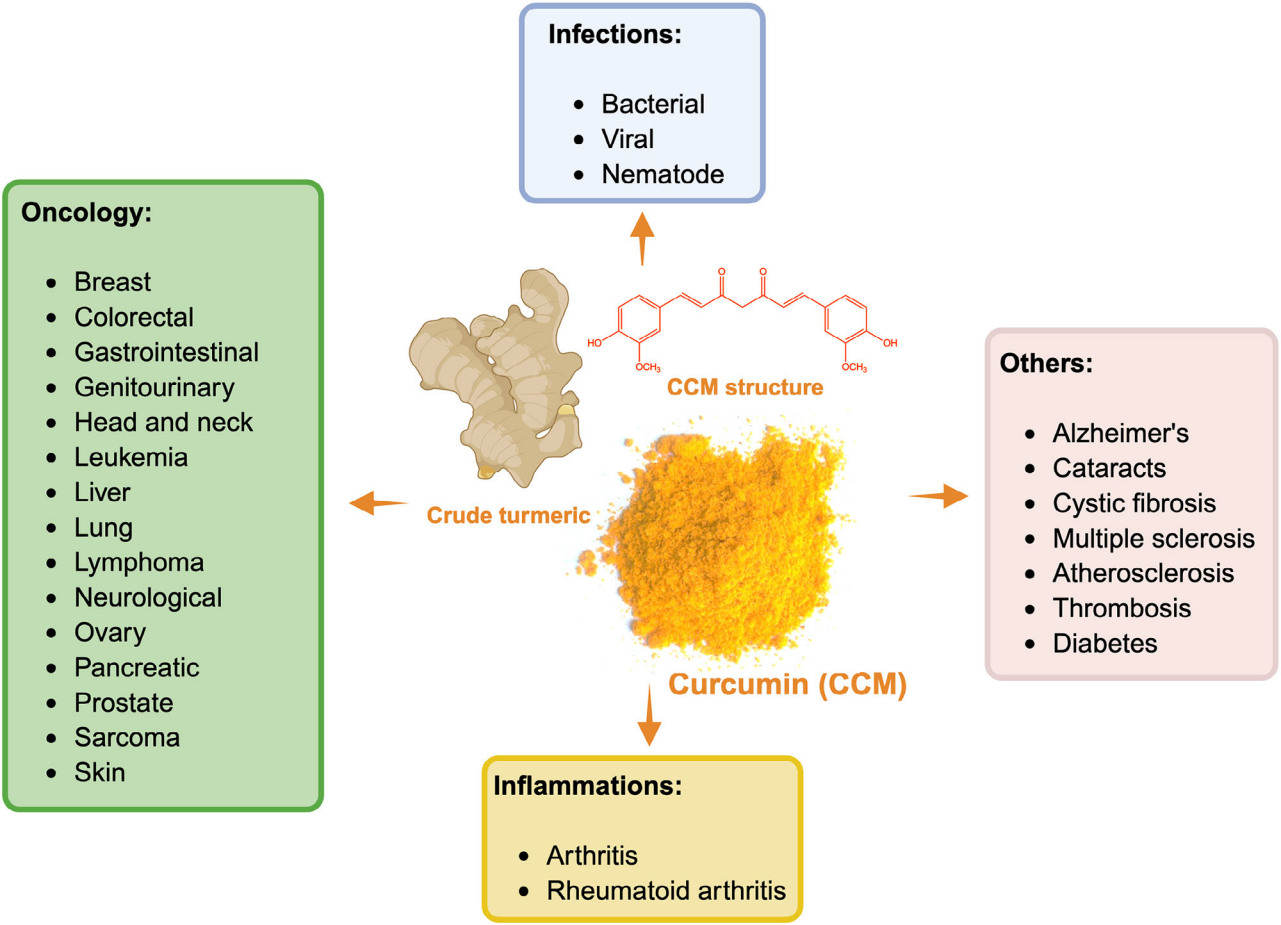
General health benefits of curcumin (CCM) in humans, adapted from Fuloria et al. [1].

Research has shown that CCM possesses strong antioxidant properties (i.e., free radical scavenging), anti‐inflammatory effects, and antimicrobial activities [1, 25, 26]. Another important area of research involving CCM is oncology. In cancer therapy, CCM has been recognized not only for its preventive potential but also for its therapeutic benefits in treating a wide range of tumors, including those affecting the gastrointestinal system, liver, prostate, breast, and lungs. Notably, CCM exhibits minimal cytotoxicity toward normal cells [15, 22]. Figure 4 illustrates the key health benefits of CCM.

Despite its immense potential, the therapeutic efficacy of free‐form CCM is significantly limited by certain limitations, including poor absorption, the inactivity of its metabolic products, and rapid elimination and clearance from the body. Although CCM's lipophilic nature enables it to penetrate cell membranes, its solubility in aqueous environments is extremely low, with a solubility value of only 5.75 µg/mL. Moreover, CCM is prone to degradation under alkaline conditions and upon exposure to light, as noted in Table 1 [22, 27].

These factors collectively reduce the bioavailability of CCM, resulting in suboptimal blood concentrations and thereby limiting its full therapeutic potential [22, 28]. To address these challenges, research in the field of nanobiotechnology has focused on developing strategies to overcome CCM's limitations [15, 22, 29]. For more detailed insights into the bioactivity and therapeutic effects of CCM, we recommend the following comprehensive works: Fuloria et al. [1], Hao and Zhang [3], Sun et al. [30], and Beganovic and Wittmann [31].

## 3 Understanding the Extraction Process: The Role of Solvents

**FIGURE 5:**
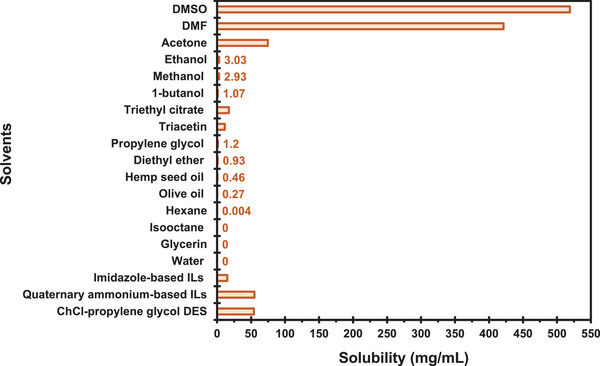
Solubility of curcumin (CCM) in various types of solvents, with values <10 mg/mL shown in the graph. Abbreviations: ChCl, choline chloride; DES, deep eutectic solvents; DMF, dimethylformamide; DMSO, dimethyl sulfoxide; and ILs, ionic liquids.

To effectively extract CCM, it is essential to understand the fundamental principles of the extraction process. Both conventional and non‐conventional methods function by disrupting plant cell walls; however, the recovery of CCM requires the use of solvents to solubilize the molecule. The choice of solvent is primarily determined by the polarity of the CCM molecule, following the principle of “like dissolves like” [32]. CCM is a hydrophobic compound, with a Log*P* value ranging from 3.2 to 4.1 (Table 1), making it nearly insoluble in water. Its solubility varies significantly depending on the solvent, with the chemical properties of each extractant influencing the degree of dissolution. For example, Tavčar and Vidak [33] investigated the solubility of CCM in various VOCs. Their results showed that polar solvents, such as dimethyl sulfoxide (DMSO) and dimethylformamide (DMF), are the most effective for dissolving CCM, with solubilities of 519.17 and 421.40 mg/mL, respectively, as illustrated in Figure 5.

These solvents exhibit strong solubilizing properties for hydrophobic compounds due to their high polarity. Acetone, while showing moderate solubility (74.70 mg/mL), is less effective than DMSO and DMF. Alcohols, such as ethanol (3.03 mg/mL), methanol (2.93 mg/mL), and 1‐butanol (1.07 mg/mL), demonstrate significantly lower solubility, making them less effective for CCM dissolution. Esters, including triethyl citrate (17.25 mg/mL) and triacetin (11.26 mg/mL), offer moderate solubility. Conversely, solvents such as propylene glycol (1.20 mg/mL), diethyl ether (0.93 mg/mL), and oils like hemp seed oil (0.46 mg/mL) and olive oil (0.27 mg/mL) exhibit very low solubility. Non‐polar solvents, including hexane (0.004 mg/mL) and isooctane (0 mg/mL), exhibit negligible solubility, highlighting the necessity of polar or semi‐polar solvents for effective CCM extraction. Hydrophilic solvents, such as glycerin and water, are also unable to dissolve CCM due to its hydrophobic nature. When selecting VOCs for CCM extraction, it is critical to evaluate the toxicological implications of the solvents used. Many conventional VOCs, particularly those with higher toxicity profiles, such as DMSO and DMF, can pose significant health and environmental risks if not handled properly. Therefore, the choice of solvents for CCM extraction should prioritize not only extraction efficiency but also compliance with safety regulations, environmental sustainability, and reduced health risks. This consideration has driven increasing interest within the scientific community toward biocompatible and sustainable solvents as alternatives to traditional VOCs. In recent years, the development and application of neoteric solvents, such as ionic liquids (ILs), deep eutectic solvents (DES), and bio‐solvents derived from renewable and biocompatible sources, have gained prominence as environmentally friendly alternatives to conventional solvents [34].

These innovative solvent systems not only enhance the solubilization of bioactive compounds like CCM but also align with sustainability goals due to their biodegradability and low toxicity. For instance, Li et al. [35] demonstrated that the aqueous solubility of CCM could reach nearly 15 mg/mL with imidazole‐based ILs, and an impressive 55 mg/mL with quaternary ammonium‐based ILs. This solubility is significantly higher than that achieved with ethanol, highlighting the superior efficiency of ILs in solubilizing hydrophobic compounds. Similarly, research using DES has yielded promising results. Le et al. [36] reported that a choline chloride (ChCl)–propylene glycol DES achieved the highest CCM recovery yield, reaching 54.2 mg/g, 1.31 times higher than the yield obtained using methanol as the solvent. These findings demonstrate the enhanced extraction efficiency of ILs and DES compared to traditional VOCs. Moreover, beyond their biocompatibility, these neoteric solvents offer improved performance in CCM extraction, representing a significant advancement in the field of green chemistry.

It is important to note that understanding the physicochemical properties of CCM and selecting appropriate solvents are essential for optimizing its extraction process, regardless of the recovery technique employed. While conventional VOCs offer well‐established performances, the emergence of neoteric solvents represents a significant advancement, particularly when sustainability and efficiency are prioritized. These innovations have the potential to enhance extraction methods, aligning them more closely with the evolving needs of the pharmaceutical, food, and cosmetic industries.

## 4 Conventional Extr action Processes

Currently, curcuminoid extraction from biological sources primarily relies on SLE, while separation and concentration are typically achieved using liquid–liquid extraction platforms that employ VOCs. Subsequent purification steps generally involve sequential chromatography techniques. However, conventional extraction processes raise significant environmental and health concerns. Many VOCs are toxic and highly flammable, posing safety risks, and exhibit low biocompatibility, which can lead to the denaturation of bioactive compounds. Moreover, the chromatographic purification of curcuminoids is inherently complex, costly, and technically challenging due to the structural similarities among CCM, DMC, and BDMC, often necessitating additional separation methods [27, 37, 38]. This complexity further increases both the cost and technical difficulty of the purification process. Despite growing awareness of these challenges within the scientific community, research in this area remains limited.

### 4.1 Maceration, Soxhlet Extraction, and Hydrodistillation

The main conventional extraction methods utilizing VOCs for CCM extraction include SLE techniques such as maceration, Soxhlet extraction, and hydro/steam distillation. While all these methods rely on VOCs to permeabilize plant cell walls and solubilize CCM, they differ in operational parameters, equipment requirements, solvent phase (liquid or vapor), and overall process complexity. The literature on these processes is extensive [39, 40]; therefore, this section does not aim to provide an exhaustive discussion but rather offers an overview of these techniques, highlighting their key characteristics, advantages, and limitations.

Maceration is a widely used solvent‐based extraction method that involves soaking plant material in a solvent at room temperature or slightly elevated temperatures for an extended period. This technique is advantageous due to its simplicity and minimal thermal degradation of heat‐sensitive compounds, such as CCM and certain VOCs. However, maceration is a slow process and often results in lower CCM yields due to inefficient mass transfer. Studies have reported CCM extraction efficiencies ranging from 2.5%–6.0% w/w using ethanol or methanol as solvents under maceration conditions lasting between 24 and 72 h. The extraction process is typically performed using a separatory funnel, which facilitates efficient phase separation of the solvent and plant extract after the soaking period. A key advantage of maceration is the high retention of VOCs, as the absence of excessive heat exposure preserves aromatic compounds such as turmerone and ar‐turmerone. However, the main limitations of this method include long processing times and relatively low extraction efficiency, making it less suitable for large‐scale applications [39, 41, 42].

Developed by Franz Ritter von Soxhlet in 1879, Soxhlet extraction is a conventional technique widely used for extracting lipophilic and poorly water‐soluble molecules. This method involves the continuous evaporation and condensation of the solvent, enabling repeated extraction cycles that maximize the recovery of the target compounds. Common VOCs used in this process include aromatic, aliphatic, and halogenated hydrocarbons, as well as alcohols, ketones, esters, and ethers. Soxhlet extraction is classified as a continuous hot extraction process, which enhances CCM recovery by improving solvent penetration and dissolution kinetics. Unlike maceration, which relies on passive diffusion, Soxhlet extraction continuously percolates fresh solvent through the solid matrix, significantly increasing extraction efficiency. Studies have shown that Soxhlet extraction using ethanol or acetone can yield up to 10%–12% w/w of CCM within 6–8 h, substantially outperforming maceration in terms of yield [39, 43, 44, 45].

However, prolonged exposure to elevated temperatures (∼60°C–80°C, depending on the solvent) poses a risk of degrading heat‐sensitive VOCs, leading to partial loss of bioactive essential oils and aroma compounds. In addition, Soxhlet extraction requires large volume of solvent, raising concerns regarding solvent recovery, environmental sustainability, and energy consumption. High temperatures (> 70°C) and extended extraction times (> 3 h) can contribute to the degradation of thermolabile molecules such as CCM, while the absence of agitation limits mass transfer, thereby reducing overall efficiency. These challenges underscore the need to optimize Soxhlet extraction or replace it with more sustainable and efficient alternatives [42, 45, 46].

Hydro/steam distillation is a conventional method primarily used for the extraction of essential oils and can also be applied to deodorize turmeric. This process is often conducted prior to the dehydration of turmeric (*C. longa*), thereby eliminating the need for a second dehydration step and improving economic feasibility. Hydrodistillation can be carried out using three main approaches: (*i*) water immersion, (ii) water immersion with vapor injection, and (iii) direct vapor injection. This versatile technique is suitable for both small‐scale and industrial applications. Hydrodistillation using the Clevenger apparatus is the standard method for analyzing volatile oils from spices and has been widely employed to extract volatile compounds from plant materials. The process involves heating a mixture of plant material and water in a distillation apparatus, which includes a condenser and a decanter to collect the condensate and separate the volatile components from *C. longa* [47, 48, 49, 50].

The separation principle of hydrodistillation relies on azeotropic distillation, wherein water molecules and oleoresin components form a heterogeneous mixture that reaches its boiling point near 100°C, despite the much higher boiling points of the oleoresin components (∼132°C–234°C). This interaction enables the mixture to distill as if it were a single compound. While hydrodistillation is primarily used for essential oil extraction, it can also facilitate CCM isolation under specific conditions. This method involves steam‐mediated extraction at temperatures typically exceeding 90°C, which volatilizes both CCM and VOCs, allowing their subsequent condensation and collection. Although effective for isolating volatile constituents such as ar‐turmerone, α‐ and β‐turmerone, and zingiberene, hydrodistillation generally yields low amounts of CCM (often below 5% w/w) due to its limited volatility and partial degradation at elevated temperatures. In addition, this process results in significant losses of thermolabile VOCs, altering the chemical profile of the extract and potentially affecting its therapeutic efficacy [42, 45, 51, 52].

Overall, while conventional extraction processes are widely accessible, simple, and relatively cost‐effective, with well‐standardized protocols, they also present significant limitations, including non‐selectivity, high energy consumption, and the use of large volumes of water and VOCs. These drawbacks are particularly concerning from both environmental and economic perspectives. Table 2 provides a comparative analysis of these methods, outlining their key characteristics, advantages, and limitations. Notably, some benefits and drawbacks are shared across multiple processes, reinforcing the need for innovative, greener extraction technologies to optimize the recovery of CCM and VOC while minimizing environmental impact.

**TABLE 2:** Comparative analysis of conventional extraction methods for curcumin (CCM): Key principles, advantages, and disadvantages.

Methods	Principle and apparatus used	Advantages	Disadvantages
**Maceration (soaking)**	Extraction based on the solubility of curcuminoids in organic solvents; typically employs a separatory funnel	Simple, low‐cost, requires minimal energy, effective for extracting thermolabile compounds, and allows a broad range of solvent choices	Long extraction time, low selectivity for non‐polar compounds, potential solvent evaporation and bioactive loss, environmental concerns from solvent disposal
**Soxhlet extraction**	Continuous solvent reflux extraction using a Soxhlet apparatus, enhancing solubilization through repeated washing	Higher CCM yield than maceration, automated and continuous processing, well‐established, enables exhaustive extraction	High solvent consumption, long extraction time, requires specialized equipment, operates at high temperatures, which may degrade thermolabile compounds
**Hydrodistillation extraction**	Steam distillation (Clevenger apparatus) to extract volatile compounds by heating the plant matrix in water	Effective for extracting volatile compounds, reduces VOC use, enables temperature control, minimizes thermal degradation compared to direct heating	Low efficiency for non‐volatile curcuminoids, high water and energy consumption, long extraction time, requires specialized equipment

## 5 Non‐Conventional (Eco‐Friendly) Extraction Processes

This refers to methods designed to increase process efficiency by reducing resource usage, such as time, energy, and solvents, or by improving extraction yields. These approaches focus on process intensification to enhance efficiency while minimizing environmental impacts. Current CCM extraction platforms pose significant environmental and health concerns, particularly due to the use of VOCs. Non‐conventional (eco‐friendly) extraction methods, including ultrasound‐assisted extraction (UAE), microwave‐assisted extraction (MAE), enzyme‐assisted extraction (EAE), and SFE, offer improved efficiency and reduced processing time. However, many of these methods still rely on VOCs, underscoring the need to not only adopt such methods but also combine them with green solvents to enhance sustainability. Eco‐friendly extraction methods, when paired with green solvents, help preserve the integrity of CCM, improve process sustainability, and minimize environmental and health risks. This combined approach aligns with the growing emphasis on eco‐efficiency in the research and production of bioactive substances. Adopting the principles of green chemistry is essential for the development of truly sustainable extraction methods. These principles include the use renewable feedstocks, the simplification of process by minimizing unnecessary derivatization, and the selection of solvents and chemicals that are biodegradable and environmentally benign. By integrating non‐conventional methods with green chemistry principles and appropriate solvent selection, researchers can achieve more efficient and sustainable extraction processes. This approach not only addresses current challenges but also promotes environmentally responsible practices in the extraction and production of natural compounds.

Although excellent studies by Jiang; Ghosh and Charcosset [39] and Manasa; Kamble and Chilakamarthi [40] have extensively reviewed CCM extraction methods from plant materials and their various applications in medicinal, biological, and food sciences, numerous studies published in recent years have further elucidated these processes or introduced innovative solutions. This review does not merely revisit eco‐friendly CCM extraction processes; rather, it aims to compare and highlights the key characteristics and outcomes of various extraction parameters, such as recovery, yield, and purity, reported over the past decade.

### 5.1 Ultrasound‐Assisted Extraction

**FIGURE 6:**
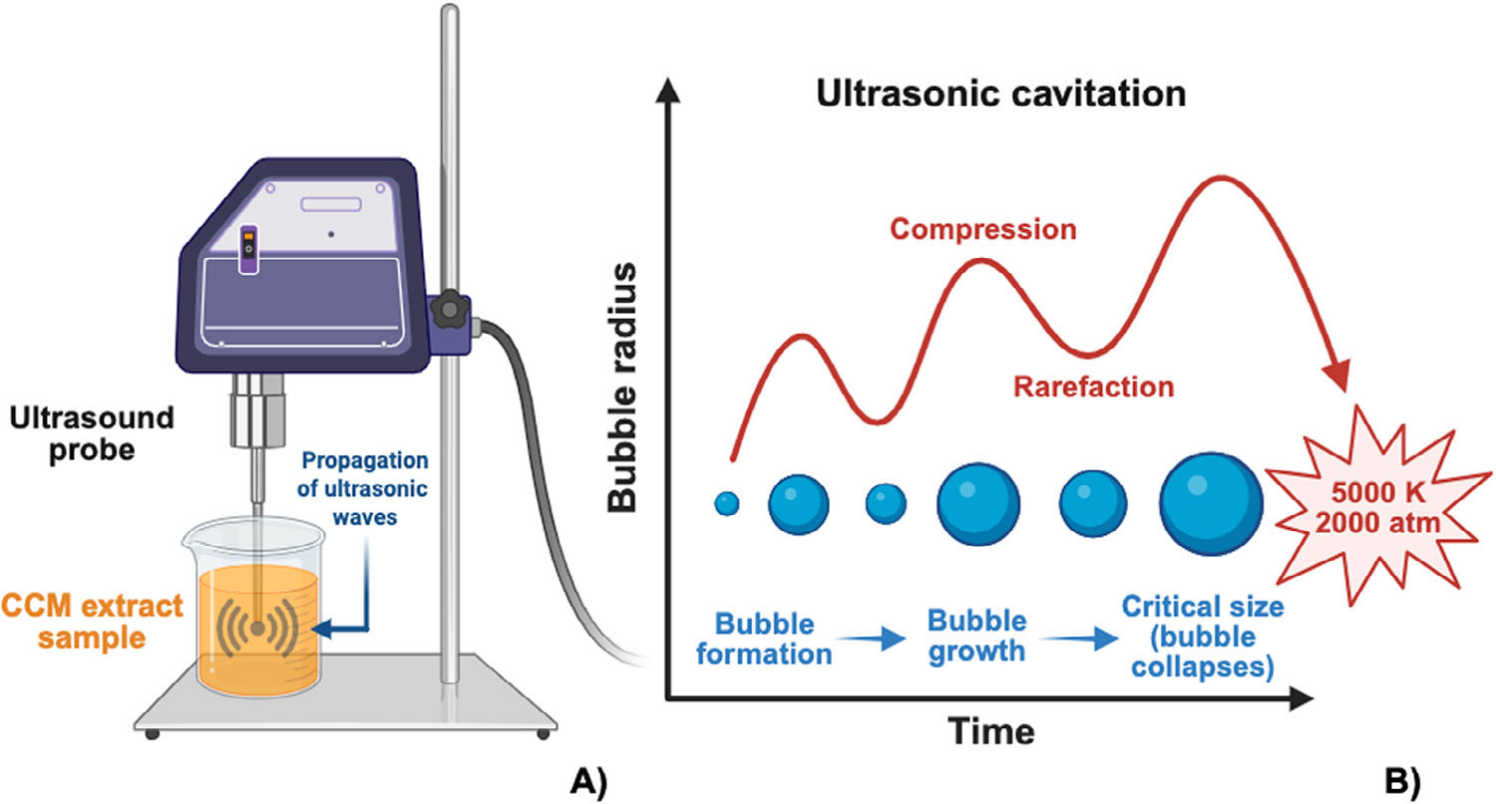
Schematic illustration of ultrasound‐assisted extraction (UAE) equipment. Ultrasonic waves enhance solvent penetration into plant cells, improving mass transfer and facilitating the release of intracellular components (**A**). During cavitation, the rapid collapse of microbubbles generates extreme localized conditions, including temperatures reaching up to 5000 K and pressures of approximately 2000 atm, with an exceptionally high cooling rate of up to 10^9^ K/s (**B**). Based on Shen et al. [53].

The ultrasound process, also known as sonication, operates based on the principle of alternating compression and expansion induced by sound waves within a frequency range of 20 kHz–100 MHz (Figure 6A). These ultrasonic waves generate microbubbles in the solution, which subsequently collapse, triggering cavitation phenomena. This process results in intense shear forces, shock waves, turbulence, mixing, and acoustic streaming (Figure 6B). The mechanical energy produced is converted into heat, supplying the activation energy required to initiate various physicochemical reactions that enhance mass transfer [53, 54]. Sonication facilitates the release of target compounds from diverse biological matrices, such as plant tissues and microbial cells, by improving mass transfer and accelerating solvent diffusion and interaction with the compounds of interest.

The efficiency of UAE is highly dependent on operational parameters such as ultrasonic frequency, temperature, extraction time, solvent properties, and the characteristics of the biological material. Therefore, optimizing these parameters is crucial to achieving a high extraction yield while preserving the integrity of bioactive compounds. Shirsath et al. [45] studied the influence of key operational factors CCM extraction using UAE. They determined that optimal extraction conditions included a temperature of 35°C, a solid‐to‐solvent ratio of 1:25 (g/mL) using ethanol, a particle size of 0.09 mm, an extraction time of 1 h, and an ultrasonic power of 250 W at 22 kHz. Under these conditions, the extraction yield reached 72%, significantly surpassing that of the maceration process, which yielded only 62% after 8 h. These results highlight UAE as a more efficient, time‐saving, and sustainable alternative to conventional methods.

Xu et al. [55] investigated UAE using hydrophilic ILs as alternative solvents for curcuminoid extraction. The study compared IL‐based UAE with ethanol‐based UAE and conventional heat–reflux extraction, which was conducted using 85% ethanol at 75°C for 4 h with a 10 mL/g liquid–solid ratio. The ILs tested included 1‐butyl‐3‐methylimidazolium bromide ([Bmim]Br), 1‐hexyl‐3‐methylimidazolium bromide ([Hmim]Br), 1‐octyl‐3‐methylimidazolium bromide ([Omim]Br), and 1‐octyl‐3‐methylimidazolium tetrafluoroborate ([Omim][BF_4_]). The researchers optimized the UAE conditions using [Omim]Br as the solvent, identifying the following optimal parameters: IL concentration of 4.2 mol/L, liquid‐to‐solid ratio of 30 mL/g, extraction time of 90 min, and ultrasonic power of 250 W. Under these conditions, the method achieved a total curcuminoid yield of 6.14%. In addition, antioxidant activity was assessed using two different assays. The 2,2'‐azinobis(3‐ethylbenzothiazoline‐6‐sulfonic acid) (or ABTS) assay resulted in 0.6244 mg Trolox equivalents (TE)/g turmeric, while the total radical‐trapping antioxidant parameter assay yielded 0.6546 mg TE/g turmeric. The study demonstrated that IL‐based UAE is a promising alternative to conventional ethanol‐based extraction, as it improved curcuminoid recovery and antioxidant activity, while simultaneously reducing extraction time and solvent consumption. These findings highlight the potential of ILs as efficient, sustainable solvents for bioactive compound extraction.

Hadi et al. [56] optimized UAE conditions for extracting curcuminoid compounds (CCM, DMC, and BDMC) from *C. domestica Val*. rhizomes. The study utilized a particle size of 0.30–0.60 mm, an extraction time of 20 min, 10 mL of aqueous extraction solvent, a temperature of 60°C, and an ultrasonic power of 55 W at 20 kHz. Under these optimized conditions, the reported extraction yields were 91.59%–98.99% for CCM, 89.79%–94.95% for DMC, and 89.33%–94.77% for BDMC. The study demonstrated significant improvements in extraction efficiency, including reduced extraction time, lower solvent consumption, and higher extraction yields, while also enhancing the overall quality of the extracts. A key finding was the successful solubilization of turmeric oleoresin in an aqueous solution through the formation of an inclusion complex with methyl‐β‐cyclodextrin. To further investigate this phenomenon, phase solubility studies were conducted using CCM as a marker compound to represent turmeric oleoresin. The results revealed that the presence of methyl‐β‐cyclodextrin significantly enhanced CCM solubility, attributed to the encapsulation effect of cyclodextrin, which improved the aqueous dispersion of the curcuminoids.

Shirsath et al. [45] compared UAE and Soxhlet extraction for CCM extraction from *C. amada*. UAE achieved a maximum yield of 72% under optimized conditions (temperature: 35°C; solid‐to‐solvent ratio: 1:25; particle size: 0.09 mm; ultrasonic power: 250 W at 22 kHz; and ethanol as the solvent), whereas Soxhlet extraction yielded only 62%. The researchers concluded that UAE was a more eco‐friendly and efficient process. In another interesting study, Liu et al. [57] developed an enhanced UAE process by incorporating graphene oxide as an auxiliary solvent for CCM extraction from *C. longa*. The study optimized key extraction parameters, including temperature (50°C), extraction time (1.5 h), ethanol concentration (60%), and graphene oxide concentration (1 mg/g), achieving a high CCM extraction yield of 64.9 mg/g. In addition, the authors conducted an economic evaluation, estimating potential savings of $1,507 per ton of turmeric under the optimized extraction conditions. They also emphasized that the lower extraction temperature and reduced ethanol consumption contribute to a safer and more environmentally friendly process, further highlighting the potential industrial applicability of this method. Further supporting the advantages of UAE, Shirsath et al. [58] investigated UAE for CCM extraction from turmeric (*C. aromatica*). The study optimized parameters, including a temperature of 40°C, a solid‐to‐solvent ratio of 1:30, a particle size of 0.09 mm from dry turmeric powder, an ultrasonic power of 240 W at 22 kHz, and ethanol as the solvent. Under these optimized conditions, UAE achieved a CCM yield of 73.18% within just 2 h, significantly outperforming conventional maceration, which required 14 h to reach a lower yield of 52.31%. The authors concluded that UAE offers superior extraction efficiency, requiring shorter processing times and lower energy consumption, while yielding higher amounts of CCM compared to conventional methods.

Unsal et al. [59] demonstrated the versatility of ILs in microextraction of CCM by applying UEA IL‐dispersive liquid–liquid microextraction to diverse food matrices, including spices, candies, and processed snacks. Their method utilized the hydrophobic affinity of 1‐butyl‐3‐methylimidazolium hexafluorophosphate ([Bmim][PF_6_]) to achieve high selectivity for CCM even in samples with high lipid content, such as margarine and potato chips. This approach not only minimized matrix interference but also maintained CCM stability during extraction, as evidenced by recovery rates of 94%–103% across all tested samples. Recent studies demonstrate that liquid‐phase microextraction methods, such as salt‐supported homogeneous liquid‐phase microextraction, also offer significant advantages. Gürmen et al. [60] employed cyclohexylamine as the extraction solvent in a saline medium to extract CCM, optimizing parameters such as pH (2.0–7.0) and NaCl mass (3 g), achieving a detection limit of 1.44 µg/L and recoveries exceeding 92% in food samples. This method, combined with spectrophotometry, proved robust against matrix interferences, reinforcing its applicability in complex matrices. In parallel, Menghwar et al. [61] developed a restricted access supramolecular solvent‐based liquid‐phase microextraction method using decanoic acid and tetrahydrofuran to form micellar phases. This system achieved an CCM detection limit of 5.3 µg/L and a preconcentration factor of 50, with recoveries of 88 to 100% in tea and supplement samples, underscoring the versatility of supramolecular solvents in extracting hydrophobic compounds. These methods share characteristics such as reduced use of VOCs, optimization of operational parameters, and integration with spectrophotometric techniques, aligning with the objectives of promoting sustainable and scalable processes.

Before implementing UAE, it is essential to thoroughly understand the behavior of the selected solvent and evaluate critical operational parameters. The physicochemical properties of the solvent, including viscosity, polarity, and thermal stability, directly influence the efficiency of the extraction process, the solubility of CCM, and solvent–matrix interactions. In addition, the ultrasonic intensity must be carefully calibrated to balance efficiency and the stability of bioactive compounds. If the applied intensity is insufficient, the extraction process will be inefficient; conversely, excessive ultrasonic energy can lead to thermal degradation of CCM, thereby reducing its bioactivity. As summarized in Table 3, the optimization of UAE parameters is crucial to achieving a high extraction yield while maintaining the structural integrity of CCM [62]. By fine‐tuning these variables, UAE can be established as a rapid, efficient, and sustainable method for CCM extraction, outperforming conventional techniques in terms of yield, processing time, and energy consumption.

**TABLE 3:** Ultrasound‐assisted extraction (UAE) parameters for curcumin (CCM): Key principles, operational conditions, and their effects on CCM extraction efficiency and stability.

**Parameters**	**Descriptions**	**Effect on CCM extraction**
**Acoustic cavitation**	Formation, growth, and collapse of microbubbles generate localized high pressure and shear forces, facilitating CCM release	Enhances CCM release by breaking plant cells and increasing mass transfer
**Microagitation**	Intensifies solvent penetration and mass transfer, enhancing CCM diffusion from plant matrices	Promotes CCM dispersion and accelerates extraction kinetics
**Mechanical effect**	Cell wall rupture and matrix disintegration improve CCM recovery by increasing the surface area available for solvent interaction	Increases yield by improving solvent access to CCM within plant tissues
**Ultrasonic frequency (kHz)**	Typically between 20–40 kHz; the optimal range depends on CCM solubility and solvent properties	Frequencies that are too high reduce cavitation efficiency, while frequencies that are too low may lead to incomplete extraction
**Ultrasonic power (W)**	Ranges from 100 to 300 W; higher power may increase extraction efficiency but can also degrade bioactive compounds	Higher power improves efficiency but must be carefully controlled to prevent degradation
**Extraction time (min)**	Optimized between 10 and 60 min; excessive exposure can cause CCM oxidation	Shorter times reduce energy consumption, while longer times may increase oxidation risks
**Solid‐to‐solvent ratio (g/mL)**	Commonly 1:20 to 1:50; higher solvent volumes enhance mass transfer but may require additional purification steps	Higher ratios improve solubility but require proper solvent recovery techniques
**Particle size (mm)**	Smaller particle sizes improve extraction yield by increasing the surface area for solvent interaction	Smaller particles allow better solvent access, enhancing extraction efficiency
**Solvent type**	Ethanol (50%–70%) is preferred due to its strong affinity for CCM, low toxicity, and eco‐friendly nature, despite being flammable	Ethanol is widely available and cost‐effective, preserving CCM integrity while reducing environmental impact
**Process temperature (°C)**	Typically controlled between 40°C–50°C to optimize extraction efficiency and prevent thermal degradation	Optimal temperatures improve CCM solubilization in the solvent phase and enhance yield without compromising bioactivity

### 5.2 Microwave‐Assisted Extraction

**FIGURE 7:**
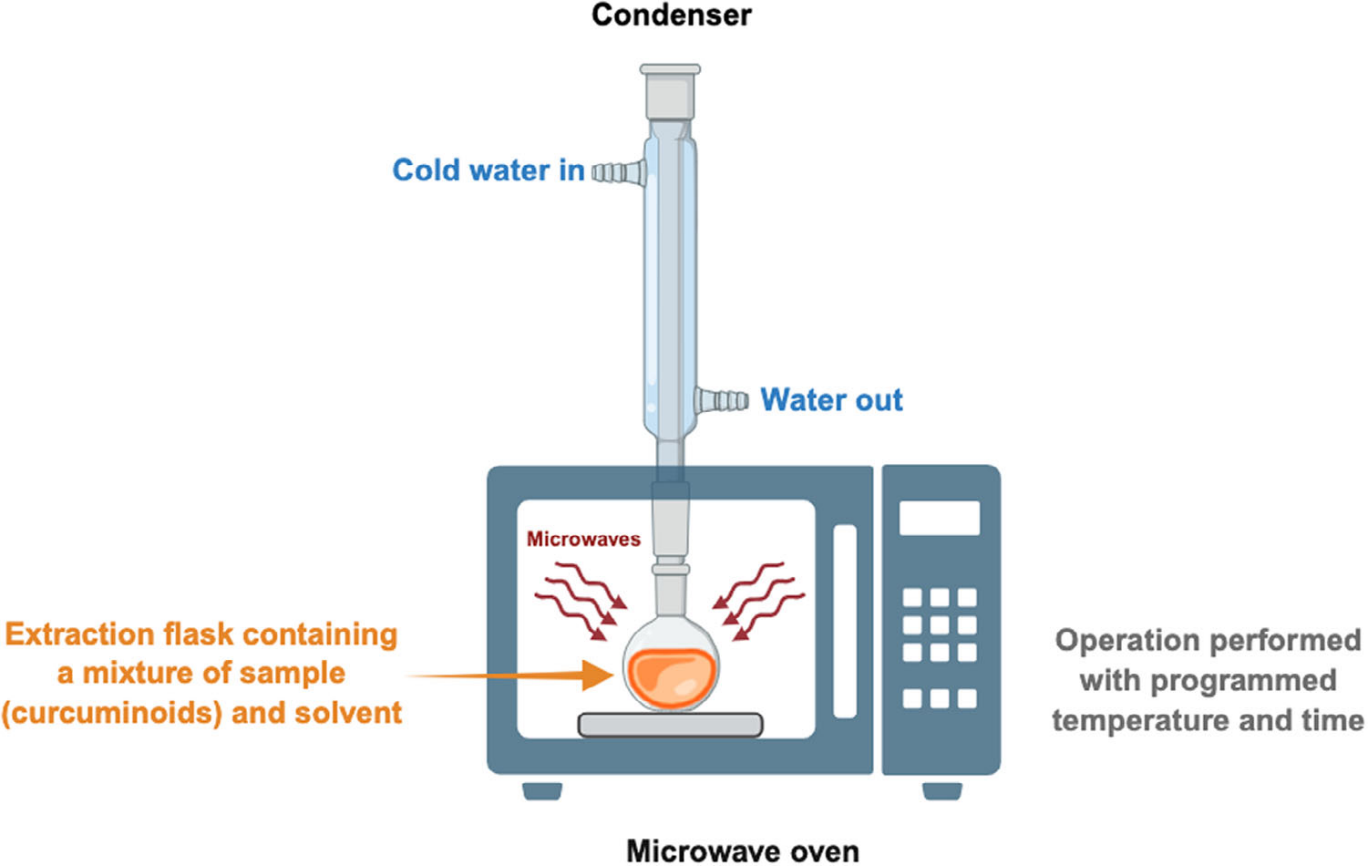
Schematic illustration of the microwave‐assisted extraction (MAE). The amount of extracted curcumin (CCM) increases rapidly at the beginning of the process and then gradually decreases until the end of extraction process.

MAE is a well‐established, automated, and sustainable technique for recovering bioactive compounds using microwave energy. This method utilizes electromagnetic waves with a frequency range of 300 MHz–300 GHz (Figure 7). MAE operates by dispersing electromagnetic waves into the extraction medium, generating heat and significantly accelerating both mass and heat transfer. This process enhances solute transport from within biological matrices into the extraction solvent, thereby improving overall extraction efficiency. Notably, MAE offers several advantages over conventional SLE methods, including reduced extraction time, lower solvent consumption, decreased energy usage, and higher extraction efficiency [54, 63].

Recent studies have evaluated and optimized various operational conditions affecting MAE efficiency for CCM extraction. Key parameters include: microwave power; extraction time (irradiation and cooling intervals); temperature; particle size of the plant material; and solvent type. In addition, process modifications such as microwave‐assisted dry irradiation (which enhances cell wall degradation) and sample pre‐treatment in powdered form (which increases susceptibility to microwave heating) can further improve both extraction rates and efficiency [64].

Hadi et al. [56] demonstrated that smaller particle sizes of dry turmeric powder (0.30–0.60 mm) increased the surface area for solvent diffusion, enhancing extraction yield. Optimized MAE conditions included 700 W of microwave energy (2.45 GHz), a 3‐min extraction time, 10 mL of solvent, and a temperature of 60°C. The study found that phenolic compound recovery increased significantly up to 3 min, but declined with longer durations due to curcuminoid degradation. Similarly, yields improved with solvent volumes up to 10 mL and temperatures up to 60°C, but decreased at higher values due to analyte decomposition. The optimized process achieved high recoveries: 92.48%–99.44% for CCM, 90.58%–97.43% for DMC, and 90.03%–96.07% for BDMC.

Bener et al. [65] observed that CCM concentration in the extract increased with MAE temperatures up to 80°C, reaching a maximum yield of 13 mg/g, but declined at higher temperatures. However, despite the degradation of CCM at elevated temperatures, the total antioxidant capacity remained stable even when the temperature was increased to 130°C. This suggests that as CCM decomposes at higher temperatures, it may generate other phenolic antioxidants, such as vanillin and ferulic acid, which help maintain antioxidant levels at the saturation value reached at 80°C. Based on these findings, 80°C and 5 min were identified as the optimal conditions for further MAE studies.

Doldolova et al. [66] investigated the recovery of curcuminoids from extracts obtained using natural deep eutectic solvents (NADES), reporting yields ranging from 37.5%–41.1%. The study emphasized the crucial role of temperature control in MAE, noting that compound solubility increases with rising temperatures. Their findings demonstrated that increasing the MAE temperature to 70°C enhanced both the total antioxidant capacity and curcuminoid content in the extracts, thereby improving overall extraction efficiency. However, the authors also cautioned that excessively high temperatures could be detrimental. Elevated temperatures may lead to the degradation of antioxidant compounds and NADES solvents, potentially compromising extraction efficiency. In addition, economic and safety concerns associated with high‐temperature processing further highlight the need for careful temperature optimization in MAE.

In another study, Fernández‐Marín et al. [67] determined the optimal MAE conditions for CCM extraction as 30 min, 160 W, and an ethanol‐to‐material ratio of 1:20 (w/v), achieving an extraction yield of 10.32%. This study emphasized that MAE significantly reduces extraction time compared to conventional methods while maintaining high yield and efficiency.

Singh et al. [68] investigated the effect of *C. longa* extracts obtained via different extraction methods on anticancer activity. The dried turmeric extract yield was highest for MAE (17.89%), followed by UAE (11.34%) and Soxhlet extraction (5.54%). In addition, MAE produced the highest concentration of bioactive compounds, with a total curcuminoid content of 326.79 mg/g, surpassing UAE (241.17 mg/g) and Soxhlet extraction (215.83 mg/g). The MAE‐derived extract exhibited strong cytotoxicity against human liver (Huh7) and colon (HCT116) cancer cell lines, demonstrating its potential for anticancer applications.

Buliga et al. [69] performed MAE using sealed vials in a Biotage Initiator microwave system, varying temperature (40°C–80°C), extraction time (5–30 min), and stirring rates (300–900 rpm). Ethanol was used as the solvent, maintaining a constant solvent‐to‐plant material ratio. The study reported significant differences in energy consumption, with MAE requiring 300 times less energy than conventional reflux extraction (using acetone and ethanol). MAE with ethanol at 900 rpm, 80°C, for 25 min showed the highest CCM content (2.73 mg/mL) and extraction efficiency of 88.6%. In addition, the energy demand for conventional extraction (reflux) was found to be 99.6% higher than MAE.

Rodsamai et al. [70] explored the use of local Southern Thai ingredients, including nipa palm syrup and nipa palm vinegar, for formulating NADES in CCM extraction. Among five NADES formulations tested, formulation D (nipa palm syrup:nipa palm vinegar:water at a 1:5:5 ratio) yielded the highest CCM content. MAE conditions were optimized using response surface methodology, with the best results obtained at a solvent ratio of 1:10, microwave power of 1000 W, and an extraction time of 51 s, resulting in 43.04 mg/g CCM recovery. The extracted CCM exhibited strong antioxidant activity and was non‐toxic to murine macrophage cell line (RAW264.7 cells) at concentrations up to 62.50 µg/mL. These findings highlight MAE combined with NADES as an eco‐friendly and efficient extraction strategy.

Before implementing MAE, it is essential to understand the solvent's physicochemical properties and the operational parameters of the microwave system. Extraction efficiency is influenced by factors such as dielectric constant, polarity, thermal stability, and microwave absorption capacity, all of which affect heating and CCM release. Optimizing key parameters, including power level, frequency, and exposure time, ensures uniform heating, prevents thermal hotspots, and enhances energy efficiency. Proper control of microwave power, extraction time, temperature, solvent selection, and sample pre‐treatment maximizes curcuminoid recovery while minimizing degradation. Future research should focus on green solvents, such as DES, to improve the sustainability of MAE‐based extractions, while also evaluating the feasibility of scaling up this process [71, 72].

### 5.3 Enzyme‐Assisted Extraction

**FIGURE 8:**
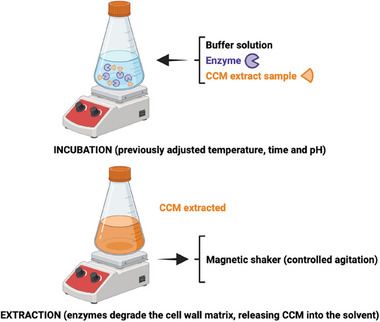
Schematic illustration of enzyme‐assisted extraction (EAE). Hydrolytic enzymes, such as α‐amylase and amyloglucosidase, are employed to degrade the structural components of cell walls. This enzymatic degradation facilitates the extraction of curcumin (CCM) from turmeric samples.

Plant cell walls are composed of complex structural polysaccharides that provide stability and resistance, posing a challenge for the extraction of intracellular components. To overcome this barrier, EAE utilizes hydrolytic enzymes to degrade the cell wall matrix, facilitating the release of bioactive compounds such as CCM into the solvent. Enzymes such as α‐amylase, glucoamylase, and amyloglucosidase are commonly employed for this purpose [42]. This method has gained attention for its efficiency, selectivity, sustainability, and eco‐friendliness. The EAE process involves adding enzymes to the solid matrix in a suitable buffer solution, followed by incubation under optimized conditions of temperature, pH, and reaction time (Figure 8). The specificity of enzymatic hydrolysis is influenced by various factors, including enzyme concentration and incubation parameters, making optimization crucial to achieving maximum extraction efficiency.

Sahne et al. [73] compared EAE, MAE, UAE, and Soxhlet extraction for CCM recovery. Soxhlet extraction achieved the highest yield (6.9%) but required large solvent volumes (350 mL), high temperatures (60°C), and prolonged extraction time (8 h), making it less sustainable. In contrast, MAE (300 W, 2 min) yielded 3.72%, UAE (35°C, 30 min) yielded 3.92%, and EAE yielded 4.1% using α‐amylase and amyloglucosidase. Optimized EAE conditions included 1 g of turmeric powder incubated in 100 mL of water, 50 mL of McIlvaine's buffer (pH 5.0), and 3% w/w of enzymes at 65°C for 6 h with 130 rpm agitation. Acetone extraction post‐enzymatic treatment further enhanced CCM diffusion. Despite taking longer than MAE or UAE, EAE preserved CCM integrity and used water as a solvent, improving environmental sustainability.

The same research group investigated the enzymatic extraction of CCM from *C. longa* using a synthesized carbamate IL (*N,N*‐dipropylammonium *N’,N’*‐dipropylcarbamate), or simply DPCARB [42]. Initially, DPCARB alone yielded 3.58% CCM at 25°C within 2 h. However, incorporating enzymatic pretreatment with α‐amylase and amyloglucosidase (4% w/w) at 65°C for 6 h improved CCM extraction to 5.73%, significantly outperforming acetone extraction (3.11% yield under identical conditions). The high purity of the extracted CCM (96%, confirmed by high‐performance liquid chromatography analysis) demonstrated the effectiveness of enzymatic pretreatment in disrupting cell walls and improving solvent diffusion. This study highlights the potential of combining enzymatic hydrolysis with advanced ILs to enhance CCM recovery while minimizing environmental impact.

Wang, Yang, and Li [74] explored a hybrid approach combining EAE with microwave and ultrasound treatments. The study tested different pectinase concentrations (0.5–8 mg/mL) over varying durations (12.5–480 min) and found that 1 mg/mL of pectinase significantly improved CCM extraction efficiency, achieving a 2.89% yield with 83.95% antioxidant activity. These authors demonstrated that enzymatic hydrolysis enhances extraction efficiency while preserving bioactivity.

Chandra et al. [75] integrated EAE with steam distillation for turmeric oil extraction. Enzymatic pretreatment using diastase, xylose, cellulase, pectinase, and lipase improved cell wall breakdown and oil release. The optimal conditions involved 180 min of enzymatic (cellulase) incubation followed by 270 min of distillation, yielding 3.75% turmeric oil rich in sesquiterpenes (e.g., 13.5%–15.6% α‐santalol, 1.2%–16.5% cineole, and 10.3%–20.7% ar‐turmerone). Although this method targeted essential oil extraction rather than CCM, it confirmed the effectiveness of EAE in enhancing bioactive compound recovery.

Patil and Rathod [76] developed an innovative enzyme co‐immobilization strategy using magnetic nanoparticles (MNPs). They immobilized glucoamylase and α‐amylase onto MNPs using glutaraldehyde (60 mM, 120 min cross‐linking time), forming spherical nanoparticles (∼100 nm) with superparamagnetic properties for easy separation. Co‐immobilized enzymes combined with low‐power ultrasound increased CCM extraction by 1.3–1.5 times. Subsequent crystallization resulted in 91% purity and a 54% yield, while immobilized enzymes retained 50% activity after 10 reuse cycles and 95% activity after 30 days of storage. This study highlights MNP‐based EAE as a scalable and reusable approach for CCM extraction.

Enzymes play a critical role in breaking down the structural components of plant matrices, facilitating the release of CCM and improving extraction efficiency. As shown in Table 4, different enzymes target specific plant cell wall components, enhancing mass transfer and solvent penetration. Among them, cellulase and pectinase effectively degrade cellulose and pectin, two major structural polysaccharides in turmeric cell walls, thereby improving CCM diffusion. Amylase breaks down starch, which otherwise acts as a barrier to solvent access. In addition, hemicellulase (xylanase) hydrolyzes hemicellulose, increasing tissue permeability and enhancing the overall extraction process. Beyond carbohydrate‐degrading enzymes, proteases such as papain hydrolyze proteins, loosening the plant matrix and facilitating CCM release. Ligninases, such as laccase and peroxidase, target lignin, a rigid polymer that can hinder solvent penetration, particularly in mature turmeric rhizomes. These enzymes work synergistically to break down plant structures, improving CCM recovery in EAE. While EAE offers advantages such as mild reaction conditions that prevent thermal degradation and preserve CCM bioactivity, it also presents challenges. High enzyme costs and extended reaction times remain key limitations compared to UAE and MAE, which operate more rapidly. However, the specificity and eco‐friendliness of enzymatic hydrolysis make it a promising approach for sustainable CCM extraction. Further research should focus on optimizing enzyme combinations, refining reaction conditions, and selecting appropriate solvents to enhance CCM recovery while ensuring an environmentally friendly and cost‐effective process. Exploring enzyme immobilization strategies and synergistic extraction techniques could also improve enzyme reusability and process scalability, making EAE a more viable option for industrial applications [74, 76, 77].

**TABLE 4:** Main enzymes employed in enzyme‐assisted extraction (EAE) of curcumin (CCM), detailing their functions, effects on extraction efficiency, and common biological sources.

**Enzymes**	**Functions**	**Effect on CCM extraction**	**Biological sources**
**Cellulase**	Breaks down cellulose in plant cell walls	Degrades plant structure, facilitating CCM release	*Trichoderma reesei*
**Pectinase**	Hydrolyzes pectin, increasing cell permeability	Enhances solvent penetration and CCM diffusion	*Aspergillus niger*
**α‐Amylase**	Breaks down starch into smaller sugars	Eliminates starchy barriers, improving CCM recovery	*Bacillus subtilis*
**Xylanase** **(hemicellulase)**	Degrades hemicellulose, enhancing tissue disruption	Increases breakdown of structural barriers, improving yield	*Trichoderma* spp.
**Protease (papain)**	Hydrolyzes proteins present in turmeric rhizomes	Softens plant tissue, enhancing CCM extraction	*Carica papaya*
**Ligninase** **(laccase and peroxidase)**	Degrades lignin, improving better solvent penetration	Enhances cell wall degradation, increasing CCM recovery	*Trametes versicolor*

### 5.4 Carbon Dioxide Supercritical Fluid Extraction

**FIGURE 9:**
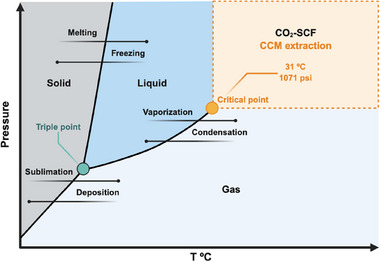
The combination of specific temperatures and pressures is used to achieve the supercritical state of carbon dioxide (CO_2_).

SFE occurs when a fluid is subjected to temperatures and pressures exceeding its critical point, acquiring both liquid‐ and gas‐like properties. In this state, the fluid exhibits a high density (similar to a liquid), low viscosity, and high diffusivity (similar to a gas), allowing it to penetrate plant matrices efficiently. The solvent power of a supercritical fluid can be tuned by adjusting temperature and pressure, making it highly selective for bioactive compound extraction. Among various supercritical fluids, carbon dioxide (CO_2_) is the preferred choice for extracting oleoresin and curcuminoids from *Curcuma* species due to its moderate critical point (31°C, 1071 psi), as highlighted in Figure 9. CO_2_ is considered a green solvent because it is non‐toxic, non‐corrosive, non‐flammable, inexpensive, abundant, and recyclable. In addition, CO_2_ can be easily separated from extracted compounds upon depressurization, eliminating the need for additional solvent removal steps and avoiding heat treatments that could degrade bioactive molecules. Processing in a CO_2_‐rich, oxygen‐free environment also minimizes oxidative degradation, preserving the quality of heat‐sensitive compounds. Due to these advantages, CO_2_‐SFE is widely applied on an industrial scale for the selective extraction of bioactive molecules [54, 78, 79, 80].

Beyond extraction, supercritical fluid chromatography has been explored for high‐purity CCM isolation. Song et al. [81] reported that this method achieved curcuminoid purities exceeding 90% in a single step, with 97.9% CCM purity, 91.1% DMC purity, and 94.8% BDMC purity, at a mean recovery efficiency of 76.6%. Supercritical fluid chromatography offers advantages such as low solvent consumption, high sample loading capacity, and efficient solvent removal, making it an attractive technique for large‐scale curcuminoid purification.

Despite its high efficiency for extracting low‐polarity compounds, CO_2_‐SFE has limited solubility for polar curcuminoids, making pure CO_2_ less effective for curcuminoid extraction. To overcome this limitation, polar co‐solvents (modifiers), such as ethanol, are added in small amounts to enhance extraction efficiency. Studies have shown that pressurized liquid extraction (PLE) with ethanol, immediately following CO_2_‐SFE, significantly improves curcuminoid recovery. Osorio‐Tobón et al. [82] demonstrated that integrating CO_2_‐SFE with PLE using ethanol resulted in curcuminoid yields of 4.3%, similar to those achieved via Soxhlet and low‐pressure solvent extraction, but with the additional benefit of recovering turmeric essential oil in the CO_2_‐SFE step. Further optimization, combining CO_2_‐SFE, PLE, and supercritical antisolvent processes, achieved 7.6% curcuminoids and 6.4% essential oil, demonstrating the economic viability of integrated extraction methods.

Solubility studies carried out by Zhan et al. [83] confirmed that temperature, pressure, and the presence of an ethanol co‐solvent significantly influenced CCM solubility in CO_2_‐SFE. Experimental measurements at 35°C, 45°C, and 55°C, under pressures of 8–20 MPa, revealed that higher pressure and temperature increased CCM solubility, while ethanol further enhanced extraction efficiency by modifying CO_2_ polarity. These findings provide valuable insights for optimizing extraction parameters to achieve maximum curcuminoid yield.

Several comparative studies have evaluated the efficacy of CO_2_‐SFE versus conventional extraction techniques. Priyanka and Khanam [84] found that Soxhlet extraction yielded 5.9% turmeric oil, whereas CO_2_‐SFE achieved 2%–5.3%, depending on process conditions. While Soxhlet extraction produced slightly higher yields, CO_2_‐SFE offered advantages such as high purity, lower solvent residue, and improved bioactivity preservation, making it economically feasible for industrial‐scale production.

Sharma, Ray and Singhal [85] optimized co‐extraction of turmeric (*C. longa*) and dried coconut shreds (*Cocos nucifera*) using CO_2_‐SFE, obtaining 45 mg/g extract at 350 bar, 65°C, and 20 min. The extracted curcuminoid content (CCM, DMC, and BDMC) reached 723 µg/g, significantly higher than achieved by Soxhlet extraction (339 µg/g after 16 h using *n*‐hexane). Slaček et al. [86] further investigated phenolic content, antioxidant activity, and curcuminoid extraction using CO_2_‐SFE, UAE, and Soxhlet extraction, demonstrating that CO_2_‐SFE yielded the highest total phenolic content (5.38 mg of gallic acid/g) and the strongest antioxidant activity [64.27% of inhibition in the 2,2‐diphenyl‐1‐picrylhydrazyl (DPPH) assay and 1750.32 mg Trolox/g dry weight in the ABTS radical cation assay]. In contrast, UAE was more effective for curcuminoid extraction (1.91 mg/g) due to enhanced mass transfer and cell wall disruption by ultrasound. In summary, while each extraction method has its own advantages, SFE stands out for its ability to extract a wide range of bioactive compounds, including phenolics, while preserving antioxidant properties. UAE, however, outperforms in selectively extracting curcuminoids, making it a preferred method for targeting specific compounds such as CCM in turmeric.

The advantages of CO_2_‐SFE extend beyond purity and efficiency, as it has been successfully combined with emerging techniques such as ultrasound‐assisted supercritical CO_2_ and NADES to further improve extraction yields. Studies indicate that ultrasonic pretreatment before CO_2_‐SFE enhances cell wall disruption, increasing solvent penetration and overall extraction efficiency. In addition, using NADES as co‐solvents has been shown to maximize curcuminoid recovery while improving bioactivity by stabilizing bioactive compounds during extraction [87, 88]. These approaches highlight the potential of CO_2_‐SFE as a highly versatile, scalable, and environmentally friendly alternative to conventional solvent‐based extraction methods. CO_2_‐SFE is recognized as a highly selective, green, and scalable method for extracting turmeric bioactives, especially when integrated with PLE, supercritical fluid chromatography, or emerging green solvents. While pure CO_2_ is highly effective for essential oil extraction, incorporating ethanol as a co‐solvent significantly enhances curcuminoid recovery, making CO_2_‐SFE a promising technique for high‐purity CCM production. Future research should focus on further optimizing co‐solvent selection, integrating process innovations, and advancing separation techniques to enhance curcuminoid purity and improve industrial feasibility. Exploring synergistic extraction methods, such as combining ultrasound‐assisted supercritical CO_2_ with NADES or integrating CO_2_‐SFE with continuous‐flow processing, may further enhance extraction efficiency and sustainability. Additional findings on CO_2_‐SFE for CCM extraction, including integrated and complementary methods, are summarized in Table 5, providing a comprehensive overview of key conditions, extraction yields, and technological advancements.

**TABLE 5:** Summary of key findings from studies on the extraction of curcumin (CCM) and curcuminoids using carbon dioxide supercritical fluid extraction (CO_2_‐SFE), with or without complementary methods. The data present the focus, key conditions, main results, and highlights from each study.

**Focus/application**	**Key conditions**	**Key results**	**Highlights**	**References**
**Optimization of CCM extraction** **from** ***C. xanthorrhiza***	T°C: 40°C; Pressure: 25 MPa; CO_2_ flow: 5.34 mL/min; Co‐solvent: ethanol	Extraction yield: 10.4% and CCM recovery: 3.2%	CO_2_‐SFE with ethanol was effective for CCM recovery. SEM analysis confirmed that the cell was disrupted by high pressure, facilitating extraction	[89]
**CO**_**2**_ **‐** **SFE with** **NADES for** **extraction of curcuminoids from** **turmeric**	Most effective extraction achieved using a menthol–lactic acid NADES as a co‐solvent, integrated at a 1:20 plant material:NADES ratio with CO_2_‐SFE	Curcuminoid yield: 33.35 mg/g. Strong antioxidant activity and enzyme inhibition; concentration‐dependent toxicity (EC_50_: 0.098 µL/mL for *Chironomus aprilinus*); high sensitivity in *Daphnia pulex*	Integrating NADES with CO_2_‐SFE maximized curcuminoid yield and enhanced bioactivity, representing a novel green extraction method	[87]
CO_2_‐SFE and SWE comparison for curcuminoids	CO_2_‐SFE [with cellulolytic enzyme mixture of 4% (v/w) Viscozyme L]; T°C: 40°C; Pressure: 35 MPa; SWE: high T°C (210°C)	CO_2_‐SFE resulted in superior CCM concentration (0.23 mg CCM/g from turmeric). SWE extracted additional compounds but degraded CCM at high T°C	CO_2_‐SFE is ideal for CCM recovery, while SWE complements extraction of other bioactives, but requires careful T°C control	[90]
**CO**_**2**_ **‐** **SFE extraction of CCM, antioxidant**s, **and lipids from turmeric**	T°C: 32°C; Pressure: 160 bar; Time: 120 min	Extraction yield: 5.88%; Total phenolic content: 155 mg GAE/g; Antioxidant activity (IC_50_): 1.34 mg/mL; CCM: 4.65 mg/L;Stru Lipids: 64.14%	CO_2_‐SFE effectively extracts antioxidants and lipids, making it suitable for health supplements, cosmetics, and commercial pharmaceutical products	[91]
CO_2_‐SFE vs. conventional solvent extraction (methanol/hexane) for turmeric bioactives	T°C: 35 to 75°C; Pressure: 75–425 bar	Optimal yield at 425 bar, 75°C. CO_2_‐SFE extracts were cleaner and free from toxic residues; stability affected by residual water	CO_2_‐SFE provided higher purity and stability compared to solvents, emphasizing its suitability for therapeutic applications	[92]
**CO**_**2**_ **‐** **SFE extraction of** **curcuminoids, oleoresins, and** **volatiles from** ***C. longa*** **and** ***C**. **amada***	*C. longa*: T°C: 65°C; Pressure: 350 bar; Time: 150 min *C. amada*: T°C: 40°C; Pressure: 300 bar; Time: 30 min Co‐solvent: 30% ethanol	*C. longa* exhibited higher antioxidant activity, *C. amada* showed better in vivo anti‐inflammatory potential	CO_2_‐SFE was promising for extracting components from two *Curcuma* species. Efficiency improved with 30% ethanol. Enzymatic pretreatment with Stargen002 (α‐amylase and glucoamylase), followed by SFE, further enhanced extraction, showcasing therapeutic applications	[93]
**Comparison of ultrasonic assisted supercritical CO**_**2**_ ***vs***. **conventional extraction (Soxhlet)** **for CCM from turmeric**	T°C: 40°C–60°C; Pressure: 15–25 MPa; Time: 30–120 min; CO_2_ flow: 2–4 mL/min; co‐solvent: 10%– 20% ethanol	Yield: 7.17% w/w; CCM: 1.69% w/w at 50°C, 25 MPa, 90 min, and 10% co‐solvent. SEM confirmed ultrasound‐enhanced cell wall disruption	Ultrasonic assisted supercritical CO_2_ significantly improved extraction efficiency over conventional methods, reducing time and enhancing yield	[88]

Abbreviations: EC_50_, concentration at which 50% of the test organisms died after 24 h of incubation; IC_50_, concentration required to achieve 50% inhibition of free radicals in the DPPH radical‐scavenging activity assay; NADES, natural deep eutectic solvent; SEM, scanning electron microscope; SWE, subcritical water extraction.

## 6 Comparative Analysis of Eco‐Friendly Extraction Methods

Over the past decade (2014–2024), research on eco‐friendly extraction methods for CCM has demonstrated distinct trends, with MAE and UAE leading in research interest, followed by SFE and EAE. Among these methods, MAE and UAE stand out with 44 and 43 publications, respectively, indicating their strong relevance in industries such as pharmaceuticals and food processing. Their advantages, including faster extraction, higher efficiency, and reduced solvent usage, likely drive their adoption. However, high equipment costs and challenges related to heat‐sensitive compounds remain significant limitations. SFE, with 36 citations, also demonstrates substantial research interest, particularly due to its selectivity and solvent‐free final products, making it ideal for high‐purity applications. However, its high energy consumption and equipment costs may have restricted its widespread use. On the other hand, EAE remains the least explored, with only 10 citations, suggesting a potential area for future development. Despite its advantages, including high selectivity, low VOC usage, and preservation of heat‐sensitive bioactives, EAE faces challenges such as complex process optimization and enzyme costs. However, as sustainability becomes a critical concern, EAE could gain traction, especially if enzyme production costs decrease. Looking ahead, UAE and MAE are expected to continue dominating in the short term, particularly as advancements in equipment design improve cost efficiency and mitigate overheating issues. Addressing SFE's cost‐related challenges could enhance its adoption in specialized applications. Meanwhile, EAE may gain momentum with advances in enzymatic technology and cost‐reduction strategies, positioning it as a promising alternative for selective curcuminoid extraction.

Table 6 summarizes the main characteristics, advantages, and limitations of these non‐conventional (eco‐friendly) extraction techniques, offering a comparative analysis based on research trends. For readers interested in further exploring this topic, we recommend the following articles: Yang et al. [94]; Chen et al. [95]; Monton et al. [96, 97]; Rahman et al. [98]; Taco et al. [99]; Christodoulou et al. [100].

**TABLE 6:** Comparative analysis of eco‐friendly extraction methods for curcumin (CCM): characteristics, advantages, and disadvantages.

**Methods**	**Principle and characteristics**	**Advantages**	**Disadvantages**	**#Published works** **(2014 to 2024)** ^a^
**Ultrasound‐assisted extraction (UAE)**	Uses high‐frequency sound waves to promote cell disintegration, facilitating the release of bioactive compounds	Fast extraction, higher yield, and efficient cell disruption leading to enhanced CCM recovery	High equipment costs and potential thermal degradation of heat‐sensitive compounds due to ultrasound‐induced heating	43 (Peak: 11 publications in 2022)
**Microwave‐assisted extraction** **(MAE)**	Uses microwave radiation to heat solvents and sample matrices, enhancing mass transfer and extraction efficiency	Reduced extraction time, higher efficiency, and lower solvent consumption	Expensive specialized equipment, industrial‐scale implementation requires reactor design, risk of overheating and compound degradation	44 (Peak: 10 publications in 2022)
**Enzyme‐assisted extraction** **(EAE)**	Uses enzymes to catalyze matrix degradation, facilitating the selective release of bioactive compounds	High selectivity, low VOC usage, and preservation of heat‐sensitive bioactives	Requires optimization of pH, temperature, and reaction time, potential interference from matrix components, high enzyme costs	10
**Supercritical fluid extraction** **(SFE)**	Uses supercritical CO_2_, which exhibits both liquid‐ and gas‐like properties, allowing selective dissolution of target compounds and extract recovery after depressurization	High selectivity, solvent‐free extracts, and adjustable operational conditions for various applications	High equipment and energy costs, limited solubility for certain compounds, requiring co‐solvents	36 (Peak: 8 publications in 2017)

^Note:^ The data were collected from the Web of Science database on December 31^st^, 2024.

^a^ The numbers were generated for the period from 2014 to 2024 using combinations of the following keywords: “curcumin” and “ultrasound‐assisted extraction”, “curcumin” and “microwave‐assisted extraction”, “curcumin” and “enzyme‐assisted extraction”, and “curcumin” and “supercritical fluid extraction”.

## 7 Conclusion and Perspectives

CCM has gained significant attention due to its diverse applications in pharmaceuticals, nutraceuticals, and cosmetics, with advancements in eco‐friendly extraction techniques playing a crucial role in enhancing its commercial viability. The continuous development of sustainable extraction technologies is expected to improve efficiency, selectivity, and scalability, ensuring higher yields while minimizing environmental impact. Computational tools such as COSMO‐SAC, COSMO‐RS, and Hansen solubility parameters are essential in enhancing efficient extraction processes by predicting solvent–solute affinity. These models aid in selecting more eco‐friendly solvents with a high affinity for CCM, thereby significantly reducing both experimental workload and solvent waste in laboratory‐scale trials for further scale‐up. It is important to note that identifying the most biocompatible solvent systems through in silico methods improves extraction selectivity and sustainability while also reducing costs and minimizing environmental impacts.

In the coming years, process intensification using UAE may benefit from advanced technologies such as controlled frequency modulation, enabling more precise targeting of plant structures to enhance extraction efficiency. MAE is expected to integrate more sophisticated temperature and power control systems, improving selectivity, reproducibility, and energy efficiency. The use of green solvents, such as ILs and NADES, in MAE is projected to expand, offering greater sustainability and improved CCM solubility. For SFE, future advancements will focus on optimizing key parameters such as pressure, temperature, and co‐solvent selection to maximize curcuminoid yield and process efficiency. Improved reactor designs and scalable extraction systems will support wider industrial adoption, particularly in high‐purity applications where solvent‐free extracts are preferred. EAE is anticipated to benefit from innovations in enzyme engineering, producing more robust and highly specific enzymes tailored for turmeric cell wall degradation. Advances in recombinant enzyme production could significantly reduce costs, making large‐scale applications more feasible. In addition, immobilized enzymes, such as those attached to MNPs or other type of nanocarriers, show promise in enhancing stability, reusability, and resistance to denaturation, ultimately improving efficiency and reducing operational costs.

A particularly promising strategy is the integration of multiple eco‐friendly extraction methods, rather than relying on a single technique. Hybrid extraction approaches, combining specific methods with appropriate green solvents, could lead to higher efficiency, reduced energy consumption, and improved product quality, making CCM extraction more sustainable and commercially viable across various bioindustries. Nevertheless, scalability, regulatory compliance, and economic feasibility continue to pose significant challenges, underscoring the necessity for pilot‐scale optimization and cost‐benefit analyses prior to full industrial implementation. It is essential to explore key sustainability pillars, such as life cycle assessment and techno–economic analysis of these nonconventional extraction techniques, to better quantify their environmental and commercial inputs. Furthermore, aligning with circular economy strategies, such as valorizing turmeric processing residues or integrating extraction processes into broader biorefinery platforms, could enhance both the sustainability and profitability of CCM production.

This review provides a comprehensive evaluation of CCM extraction methods, offering valuable insights for researchers and industry professionals seeking high‐quality, energy‐efficient, and scalable extraction solutions. While conventional methods face limitations such as long extraction times and environmental concerns, emerging green technologies offer more sustainable and efficient alternatives. Ultimately, the integration of computational tools, artificial intelligence, and machine learning has the potential to transform process optimization. By utilizing predictive modeling in each operational unit, namely, for extraction efficiency, energy consumption, and solvent interactions, it may become feasible to develop and implement real‐time control over cost‐effective, eco‐friendly extraction techniques. These advancements will not only ensure the large‐scale, sustainable production of CCM but also set the stage for similar methods in extracting other high value bioactives compounds, thereby fostering a greener, smarter, and more resilient bioeconomy.

## Author Contributions

**Isabelle O. Torquato**: methodology, formal analysis, writing – original draft. **Astrid Corrales**: methodology, formal analysis, writing – original draft. **Cassamo U. Mussagy**: writing – review and editing. **Jorge F. B. Pereira**: writing – review and editing. **André M. Lopes**: conceptualization, project administration, supervision, writing – review and editing.

## Conflicts of Interest

The authors declare no conflicts of interest.

## Data Availability

The data that support the findings of this study are available from the corresponding author upon reasonable request.
